# Structure of *Cryptosporidium* IMP dehydrogenase bound to an inhibitor with *in vivo* antiparasitic activity

**DOI:** 10.1107/S2053230X15000187

**Published:** 2015-04-21

**Authors:** Youngchang Kim, Magdalena Makowska-Grzyska, Suresh Kumar Gorla, Deviprasad R. Gollapalli, Gregory D. Cuny, Andrzej Joachimiak, Lizbeth Hedstrom

**Affiliations:** a Center for Structural Genomics of Infectious Diseases, Computational Institute, University of Chicago, 5735 S. Ellis Avenue, Chicago, IL 60637, USA; b Structural Biology Center, Biosciences, Argonne National Laboratory, 9700 S. Cass Avenue, Argonne, IL 60439, USA; c Department of Biology, Brandeis University, 415 South Street, Waltham, MA 02454, USA; d Department of Pharmacological and Pharmaceutical Sciences, College of Pharmacy, University of Houston, Science and Research Building 2, Houston, TX 77204, USA; e Department of Chemistry, Brandeis University, 415 South Street, Waltham, MA 02454, USA

**Keywords:** *Cryptosporidium*, inosine 5′-monophosphate dehydrogenase, P131

## Abstract

Inosine 5′-monophosphate dehydrogenase (IMPDH) is a promising target for the treatment of *Cryptosporidium* infections. Here, the structure of *C. parvum* IMPDH (*Cp*IMPDH) in complex with inosine 5′-monophosphate (IMP) and P131, an inhibitor with *in vivo* anticryptosporidial activity, is reported. P131 contains two aromatic groups, one of which interacts with the hypoxanthine ring of IMP, while the second interacts with the aromatic ring of a tyrosine in the adjacent subunit. In addition, the amine and NO_2_ moieties bind in hydrated cavities, forming water-mediated hydrogen bonds to the protein. The design of compounds to replace these water molecules is a new strategy for the further optimization of *C. parvum* inhibitors for both antiparasitic and antibacterial applications.

## Abstract 2

Compound P131 displays antiparasitic activity in a mouse model of *Cryptosporidium* infection, validating IMP dehydrogenase (IMPDH) as a drug target. Here, the structure of the enzyme–substrate–P131 complex is reported at 2.05 Å resolution. The structure is an important step to further refine the design of IMPDH inhibitors.

## 1. Introduction   

*Cryptosporidium* parasites are protozoan pathogens of increasingly recognized importance in both the developing and the developed world (Striepen, 2013 ▶). Cryptosporidiosis is a major cause of morbidity in infants and malnutrition in young children in Africa and Asia, and is a major cause of waterborne disease in North America and Europe. Infections are self-limiting in immunocompetent individuals, but can be chronic and fatal in immunocompromised patients (Checkley *et al.*, 2014 ▶). In addition, *Cryptosporidium* oocysts are resistant to commonly used methods of water treatment and are readily available, making this organism a potential weapon for bioterrorism. The potential damage of such an incident was illustrated in the naturally occurring outbreak in Milwaukee in 1993, where 400 000 cases were reported and an estimated $96 million in damages were incurred.

Nitazoxanide is the only FDA-approved drug for the treatment of cryptosporidiosis (Checkley *et al.*, 2014 ▶). It has limited efficacy in immunocompetent individuals and no efficacy in immunocompromised patients. High doses of paromomycin have anticryptosporidial activity in mice, where nitazoxanide is not effective (Tzipori *et al.*, 1994 ▶; Baishanbo *et al.*, 2006 ▶), but has not proved effective in humans (Hewitt *et al.*, 2000 ▶). Thus, there is a critical need for new chemotherapy to treat *Cryptosporidium* infections.

*Cryptosporidium* parasites have a very streamlined purine-biosynthetic pathway that relies on the salvage of adenosine from the host (Striepen *et al.*, 2004 ▶). Adenosine is converted into guanine nucleotides in a process that requires IMP dehydrogenase (IMPDH) activity. Curiously, the *Cryptosporidium* gene for IMPDH appears to have been obtained from bacteria *via* lateral transfer, so that the parasite enzyme is very different from the host ortholog. IMPDH is a clinically validated target for immunosuppressive, anticancer and antiviral therapy (Hedstrom, 2009 ▶). The IMPDH reaction involves two chemical transformations (Hedstrom, 2009 ▶). The first step is nucleophilic attack of the active Cys219 at the C2 position of IMP, followed by hydride transfer to form the covalent intermediate E-XMP* and NADH. The reduced cofactor dissociates from the enzyme and the mobile active-site flap (residues 302–330) folds into the cofactor site, positioning the catalytic Arg315 to activate water for the hydrolysis of E-XMP* to yield the product xanthosine 5′-monophosphate (XMP). IMPDH is a homotetramer with square-planar symmetry. The four active sites are located near the subunit interfaces. The IMP site is contained within a monomer, and the residues that contact IMP are strongly conserved among all IMPDHs (Hedstrom, 2009 ▶). The nicotinamide portion of the cofactor site also lies within the same monomer as IMP, and is also strongly conserved, as expected given that this is the site of chemical transformation. However, the position of the adenosine portion of the cofactor site varies widely among IMPDHs from different organisms. The adenosine site of eukaryotic IMPDHs is largely within the same monomer as the IMP site (Colby *et al.*, 1999 ▶; Prosise & Luecke, 2003 ▶), but in prokaryotic IMPDHs the adenosine portion of NAD^+^ binds in a pocket in the adjacent subunit (Makowska-Grzyska *et al.*, 2015 ▶).

**Figure 1:**
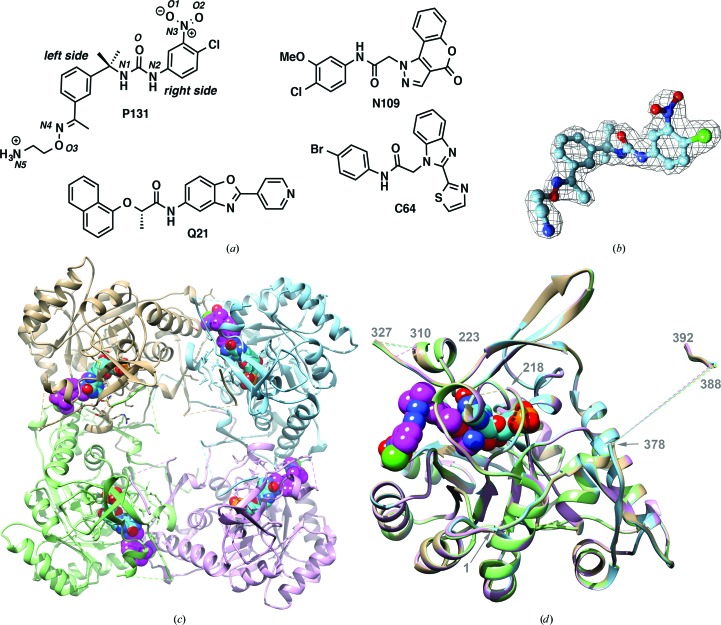
(*a*) Structure of P131 and other cpIMPDH inhibitors. The numbering scheme is shown in italics. (*b*) 2*mF*_o_ − *DF*_c_ electron-density map for P131 contoured at the 1σ level. The inhibitor is shown in ball-and-stick representation. (*c*) Structure of the *Cp*IMPDH tetramer. Subunit *A* is in tan, subunit *B* is in light blue, subunit *C* is in pink and subunit *D* is in light green. IMP and P131 are shown as spheres with the following color coding: carbon, cyan for IMP and magenta for P131; oxygen, red; nitrogen, blue; phosphorus, orange; chlorine, green. (*d*) Superposition of the individual monomers. Dashed lines indicate disordered regions. This figure was produced with *UCSF Chimera* (Pettersen *et al.*, 2004 ▶).

We have been engaged in a program to design selective inhibitors of *C. parvum* IMPDH (*Cp*IMPDH) as potential anticryptosporidial agents (Gorla *et al.*, 2012 ▶, 2013 ▶, 2014 ▶; Johnson *et al.*, 2013 ▶; Kirubakaran *et al.*, 2012 ▶; MacPherson *et al.*, 2010 ▶, Maurya *et al.*, 2009 ▶; Sharling *et al.*, 2010 ▶; Sun *et al.*, 2014 ▶; Umejiego *et al.*, 2004 ▶, 2008 ▶). Compound P131 is the most promising compound to arise from this effort (Fig. 1 ▶ *a*), with superior activity to nitazoxanide and paromomycin in a mouse model of acute disease (Gorla *et al.*, 2014 ▶).

The prokaryotic origin of *Cp*IMPDH suggested that these inhibitors might also be effective against IMPDHs from pathogenic bacteria (Gollapalli *et al.*, 2010 ▶). The *Cp*IMPDH inhibitor collection does contain many potent inhibitors of *Bacillus anthracis* IMPDH, but only a few of these compounds possess antibacterial activity (Mandapati *et al.*, 2014 ▶). The *Cp*IMPDH inhibitors are generally more hydrophobic, with less polar surface area, than antibiotics (O’Shea & Moser, 2008 ▶), which suggests that they do not efficiently enter bacteria. Enzyme–inhibitor structures provide important information for the further modification of *Cp*IMPDH inhibitors both for antiparasitic and antibacterial activities.

Here, we report the structure of the enzyme–IMP–P131 complex. This information will facilitate the further optimization of *Cp*IMPDH inhibitors for the treatment of crypto­sporidiosis.

## 2. Materials and methods   

Unless otherwise noted, all reagents and solvents were purchased from commercial sources and were used without further purification. Compound P131 was synthesized as described previously (Gorla *et al.*, 2012 ▶).

### 2.1. Protein production and crystallization   

The coding sequence for *Cp*IMPDH was amplified by PCR and cloned into pMCSG7 vector (Stols *et al.*, 2002 ▶) as described in Gorla *et al.* (2013 ▶). The recombinant construct produced a fusion protein with an N-terminal His_6_ tag and a TEV protease recognition site (ENLYFQ↓S) (Table 1 ▶). The fusion protein was expressed in *Escherichia coli* strain BL21(DE3) harboring the pMAGIC plasmid encoding one rare *E. coli* Arg tRNA (covering the codons AGG/AGA) in the presence of 100 µg ml^−1^ ampicillin and 30 µg ml^−1^ kanamycin (Kim *et al.*, 2011 ▶). The cells were grown in enriched M9 medium at 37°C followed by overnight induction with 0.5 m*M* isopropyl β-d-1-thiogalactopyranoside (IPTG) at 18°C. Cells were harvested, resuspended in lysis buffer (50 m*M* HEPES, pH 8.0, 500 m*M* KCl, 20 m*M* imidazole, 1.5 m*M* TCEP, 5% glycerol) and stored at −80°C. *Cp*IMPDH protein was purified according to a standard protocol (Makowska-Grzyska *et al.*, 2014 ▶). The protocol included cell lysis by sonication and nickel-affinity chromatography on an ÄKTAxpress system (GE Healthcare Life Sciences) followed by His_6_-tag cleavage using recombinant TEV protease (Blommel & Fox, 2007 ▶) and additional Ni^2+^-affinity chromatography to remove the protease, the uncut protein and the affinity tag. In the final step, the protein was dialyzed against crystallization buffer consisting of 20 m*M* HEPES pH 8.0, 150 m*M* KCl, 1.5 m*M* TCEP, concentrated, flash-cryocooled and stored in liquid nitrogen. Crystallizations were set up with the help of a Mosquito liquid dispenser (TTP LabTech). Protein samples used for crystallization consisted of 13.4 mg ml^−1^ protein in crystallization buffer (as above), 5 m*M* IMP, 1.5 m*M* P131. Diffraction-quality crystals appeared at 16°C in 0.1 *M* Bicine–NaOH pH 9.0, 20%(*w*/*v*) PEG 6000 (Table 2 ▶). The crystals were mounted on CryoLoops (Hampton Research) and flash-cooled in liquid nitrogen. The cryoprotectant consisted of 0.1 *M* Bicine–NaOH pH 9.0, 20%(*w*/*v*) PEG 6000, 15% glycerol.

### 2.2. Data collection and structure determination   

Diffraction data were collected at 100 K on the 19-ID beamline of the Structural Biology Center (SBC) at the Advanced Photon Source (APS), Argonne National Laboratory (Rosenbaum *et al.*, 2006 ▶). Data were collected at a single wavelength of 0.97931 Å (12.6603 keV) to 2.05 Å resolution from a single crystal of *Cp*IMPDH complexed with IMP and P131. The crystal was exposed for 3 s per 1.0° rotation in ω at a crystal-to-detector distance of 300 mm. The data were recorded on an ADSC Q315r CCD detector scanning 190°. The *SBC-Collect* program was used for data collection and visualization. Data collection, integration and scaling were performed with the *HKL*-3000 program package (Minor *et al.*, 2006 ▶). A summary of the crystallographic data is presented in Table 3 ▶.

The structure was determined by molecular replacement using the structure of *Cp*IMPDH (PDB entry 3ffs; MacPherson *et al.*, 2010 ▶) as a search model with the *HKL*-3000 suite using data to 3.0 Å resolution (Bricogne *et al.*, 2003 ▶; Minor *et al.*, 2006 ▶). The initial model contained four protein chains of the search model, and there was extra electron density for additional protein residues that were not part of the search model. The presence of IMP and P131 in the active site was apparent from the initial electron-density map (*F*_o_). Extensive manual model building with *Coot* (Emsley & Cowtan, 2004 ▶) and subsequent refinement using *phenix.refine* (Bricogne *et al.*, 2003 ▶; Afonine *et al.*, 2012 ▶) were performed against the full data set to 2.05 Å resolution until the structure converged to an *R* factor (*R*_work_) of 0.187 and an *R*_free_ of 0.229 with an r.m.s.d. on bond distances of 0.004 Å and an r.m.s.d. on bond angles of 0.864°. The asymmetric unit contained four protein chains (*A*, *B*, *C* and *D*), with residues 1–392 for all chains and a Ser-Gly-Gly linker inserted in place of residues 90–134 (MacPherson *et al.*, 2010 ▶). Several N- and C-terminal amino acids, including the N-terminal Ser-Asn-Ala introduced during cloning (Eschenfeldt *et al.*, 2010 ▶), were missing because of disorder. In addition, several residues within the active-site flap were disordered and were not modeled. These included residues 311–324 in chains *A*, *B* and *D* and 310–325 in chain *C*. The final model also contained four IMP molecules, four P131 molecules, five ethylene glycol molecules, one glycerol molecule and 340 ordered water molecules. The stereochemistry of the structure was checked with *PROCHECK* (Laskowski *et al.*, 1993 ▶) and a Ramachandran plot. Refinement statistics are shown in Table 4 ▶. Atomic coordinates and experimental structure factors of the structure have been deposited in the PDB as entry 4rv8.

## 3. Results and discussion   

The structure of *Cp*IMPDH in complex with IMP and P131 was solved at 2.05 Å resolution using molecular replacement with the structure of apo *Cp*IMPDH (PDB entry 3ffs) as the search model (Table 4 ▶). Density for P131 is clearly visible in all four subunits (Fig. 1 ▶ *b*). The unit cell contains a single tetramer with *D*4 symmetry (Fig. 1 ▶ *c*). The four subunits are nearly identical, with r.m.s.d.s ranging between 0.15 Å (chain *A* *versus* chains *B*, *C* and *D*) and 0.16 Å (chain *C* *versus* chains *B* and *D*) (Fig. 1 ▶ *d*). Residues 311–324, which are part of the active-site flap (see below), and residues 378–387 near the C-terminus are disordered in all four subunits. In addition, residues 310 and 325 are disordered in chain *C*.

**Figure 2:**
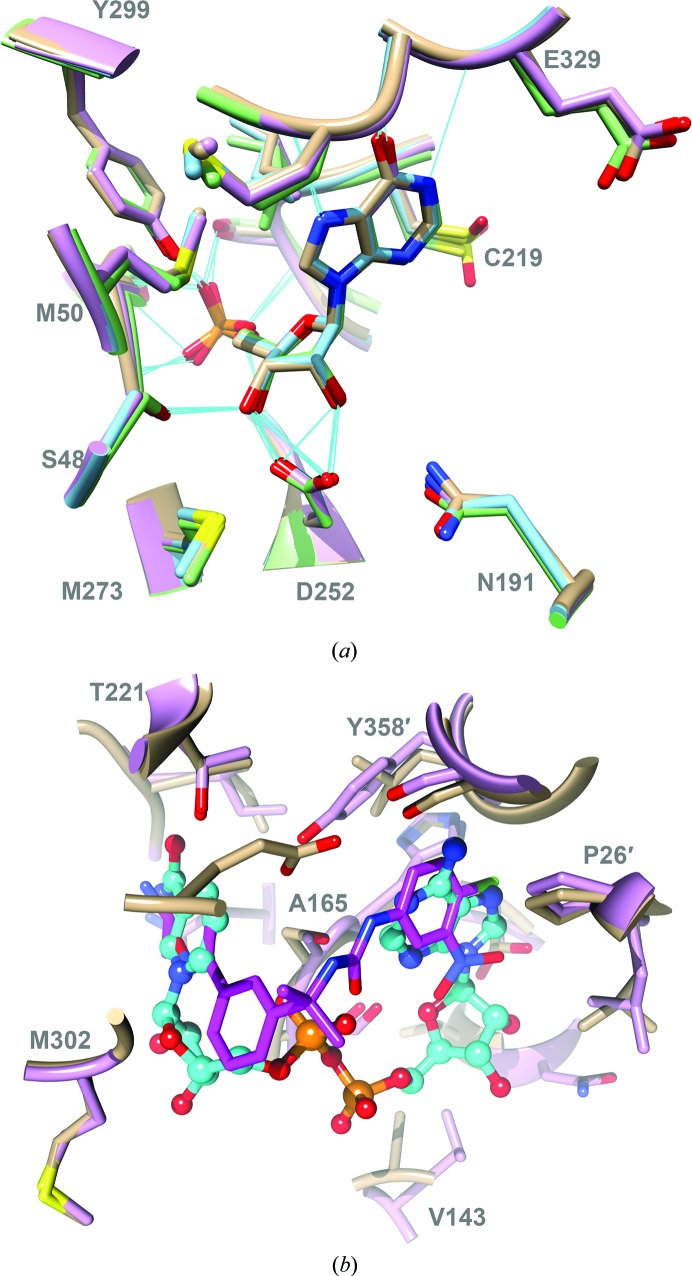
Substrate-binding and cofactor-binding sites. (*a*) The IMP site of *Cp*IMPDH in complex with inhibitors. Residues in contact with IMP are shown. P131, PDB entry 4rv8 (pink); C64, PDB entry 3khj (green; MacPherson *et al.*, 2010 ▶); Q21, PDB entry 4ixh (blue; Gorla *et al.*, 2013 ▶); N109, PDB entry 4qj1 (tan; Center for Structural Genomics of Infectious Diseases, Sun *et al.*, 2014 ▶). The *B* subunit is shown in all four cases. (*b*) Superposition of the *Cp*IMPDH–IMP–P131 complex (*AD* dimer, pink) with the IMP–NAD^+^ complex of *V. cholerae* IMPDH (*AA* dimer, tan; PDB entry 4qne; Center for Structural Genomics of Infectious Diseases, Makowska-Grzyska *et al.*, 2015 ▶). P131 is shown in stick representation with C atoms in magenta. NAD^+^ is shown in ball-and-stick representation with C atoms in cyan. The residues that contact NAD^+^ are shown, with the exceptions of IMP, Cys219 and Met302-Gly303, which were omitted for clarity (*Cp*IMPDH numbering). This figure was produced with *UCSF Chimera* (Pettersen *et al.*, 2004 ▶).

The conformation and interactions of IMP in the P131 complex are essentially identical to those observed in the previously reported complexes of *Cp*IMPDH with C64, Q21 and N109 (Fig. 2 ▶ *a*; MacPherson *et al.*, 2010 ▶; Gorla *et al.*, 2013 ▶; Sun *et al.*, 2014 ▶), as well as those in the complexes of *Bacillus anthracis*, *Campylobacter jejuni* and *Clostridium perfringens* IMPDHs with various *Cp*IMPDH inhibitors (Makowska-Grzyska *et al.*, 2015 ▶).

**Figure 3:**
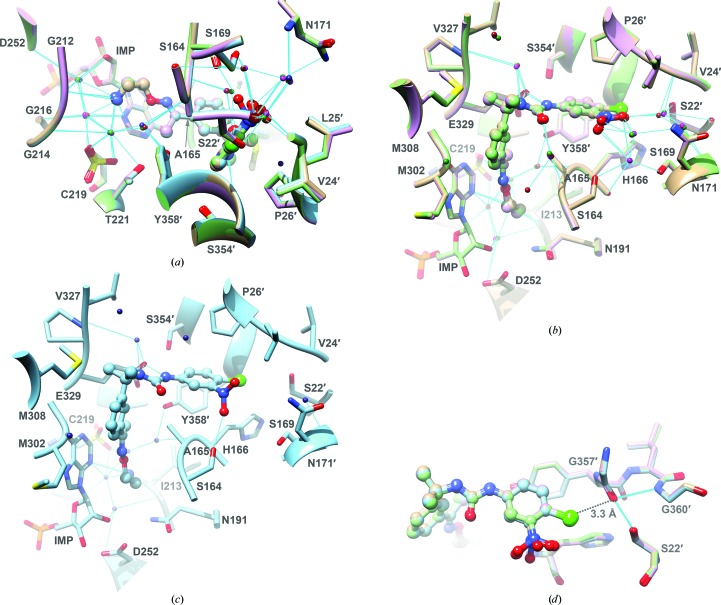
Interactions of P131 with *Cp*IMPDH. (*a*) Interactions of the oxime and alkylamine. P131 is displayed in ball-and-stick representation and the protein residues are in ribbon representation with side chains depicted as sticks. Hydrogen bonds are shown in cyan. The dimer formed by subunits *A* and *D* is shown in tan and the corresponding waters are shown in brown. The *BA* dimer is shown in light blue and the corresponding waters are shown in dark blue. The *CB* dimer is shown in pink and the corresponding waters are shown in magenta. The *DC* dimer is shown in light green and the waters are shown in dark green. Residues within 5 Å of P131 are shown, with the exceptions of Asn191, residues 327–329 and the side chains of His166, Ile213 and Asn252, which have been omitted for clarity. (*b*) Interactions of the urea and the right side of P131 in the *AD*, *CB* and *DC* dimers. The color scheme is as above. (*c*) Interactions of the urea and the right side of P131 in the *BC* dimer. The color scheme is as above. (*d*) Interaction of the P131 Cl atom with the carbonyl O atom of Gly357′. The color scheme is the same as above. Hydrogen bonds are shown in cyan. The halogen bond is shown as a black dashed line. This figure was produced with *UCSF Chimera* (Pettersen *et al.*, 2004 ▶).

As observed with other *Cp*IMPDH inhibitors, P131 binds in the cofactor site, spanning both the nicotinamide and adenosine subsites (Fig. 2 ▶ *b*). The left-side aromatic ring (Fig. 1 ▶ *a*) binds in the nicotinamide subsite, which is a pocket formed by IMP and residues Ser164-Ala165, Asn191, Gly212–Gly214, the catalytic Cys219, Thr221, Met302-Gly303, Met308, Glu329 and Tyr358′ (where a prime denotes a residue from the neighboring subunit). The electron density indicates that Cys219 has oxidized to the sulfenic acid, as often occurs with reactive cysteine residues. Similar oxidation of the catalytic Cys was observed in the structures of *Tritrichomonas foetus* IMPDH (Prosise & Luecke, 2003 ▶; Prosise *et al.*, 2002 ▶). Despite this modification, the position of Cys219 is very similar to that observed in other *Cp*IMPDH–inhibitor complexes (Fig. 3 ▶ *a*). The oxime group of P131 lies across the hypoxanthine ring of IMP, while the N5 amine forms hydrogen bonds to the main-chain carbonyl O atom of Gly212 and N3 of IMP in all of the subunits (Fig. 3 ▶). The amine also interacts with water molecules to create a hydrogen-bonding network that extends to the 2′-OH of IMP, the sulfenic acid of Cys219, Ala165 N, Gly212 O, Gly214 N, Thr221 OG, Asp252 O and Tyr358 OH′. While the protein and inhibitor conformations are nearly identical in this region in all four subunits, the water occupancy varies slightly. WAT213, WAT282 and WAT316 (numbering in chains *A*, *B* and *D*, respectively) form hydrogen bonds to Tyr358 OH′ and Ala165 O in chains *A*, *B* and *D*, but this water is absent in chain *C* (Fig. 2 ▶ *b*). Curiously, despite these many interactions, the presence of the alkylamine decreases the inhibitor potency by a factor of ∼30 *versus* the corresponding oxime (IC_50_ = 0.66 n*M* for P96 *versus* 20 n*M* for P131; Gorla *et al.*, 2012 ▶).

Also as observed with other *Cp*IMPDH inhibitors (MacPherson *et al.*, 2010 ▶; Gorla *et al.*, 2013 ▶; Sun *et al.*, 2014 ▶), P131 bends in the linker region so that the plane of the right-side aromatic moiety is almost perpendicular to the left-side aromatic moiety (Figs. 1 ▶ *d*, 3 ▶ *b* and 3 ▶ *c*). Both urea N atoms of P131 form hydrogen bonds to Glu329 OE2 in all four subunits (Fig. 3 ▶ *b* and 3 ▶ *c*). Analogous interactions with Glu329 are observed with other *Cp*IMPDH inhibitors. In addition, water-mediated hydrogen bonds are found between the urea N1 atom and Val327 O as well as between the urea carbonyl O atom and Ala165 N.

The right-side aromatic ring binds in the adenosine subsite formed by residues Ser22′, Val24′-Leu25′-Pro26′, Ser164-Ala165-His166, Ser354′ and Gly357′-Tyr358′ (Makowska-Grzyska *et al.*, 2015 ▶). As noted above, this subsite is highly divergent, accounting for the selectivity of the *Cp*IMPDH inhibitors. The analogous residues in human IMPDH type 2 are Ile42′, Phe44′-Thr45′-Ala46′, Ser275-Ser276-Gln277, His466 and Gln469′-Asp470′. As observed with the primary amine, the NO_2_ group binds in a hydrophilic pocket. In subunits *A*, *C* and *D*, the NO_2_ group is roughly parallel to the aromatic ring and forms water-mediated hydrogen bonds to Ser22 O′, Val24 O′, Ser164 OG, His166 O, Ser169 O and Asn171 N and ND2. In contrast, the NO_2_ group is perpendicular to the ring in subunit *B* and forms a hydrogen bond directly to Ser164 OG (Figs. 2 ▶ *c* and 2 ▶ *d*).

The P131 Cl atom interacts with the carbonyl O atom of Gly357′ (Fig. 3 ▶ *d*; average distance 3.33 ± 0.12 Å), which suggests the presence of a halogen bond (expected distance 3.0–3.40 Å; Bissantz *et al.*, 2010 ▶). The C—Cl⋯O and Cl⋯O—C angles are compatible with halogen-bond formation with the π system of the carbonyl acting as the donor (128 and 82°, respectively, *versus* C—Cl⋯O ≥ 120° and 180° ≥ Cl⋯O—C ≥ 70°; Bissantz *et al.*, 2010 ▶; Voth *et al.*, 2009 ▶; Vallejos *et al.*, 2012 ▶). The Gly357′ carbonyl is further polarized by hydrogen bonds to Ser22 OG′ (average distance 2.85 ± 0.06 Å) and Gly360 N′ (average distance 3.19 ± 0.06 Å). The Gly357′–Ser22 OG′ hydrogen bond is at approximately 90° relative to the halogen bond, similar to the angle between other pairs of halogen and hydrogen bonds.

The water-mediated hydrogen-bonding networks of both the left-side alkylamine and the right-side NO_2_ group offer many opportunities for further inhibitor optimization. In particular, the repurposing of *Cp*IMPDH inhibitors as antibacterial agents requires the introduction of additional polarity (Mandapati *et al.*, 2014 ▶), which might be attained by extending the inhibitor to replace water in these networks. Efforts in this area are ongoing.

## Supplementary Material

PDB reference: *Cp*IMPDH–IMP–P131, 4rv8

## Figures and Tables

**Table 1:** Protein-production information

Source organism	*C. parvum* Iowa II
CryptoDB ID	cgd6_20
CryptoDB sequence	MGTKNIGKGLTFEDILLVPNYSEVLPREVSLETKLTKNVSLKIPLISSAMDTVTEHLMAVGMARLGGIGIIHKNMDMESQVNEVLKVKNWISNLEKNESTPDQNLDKESTDGKDTKSNNNIDAYSNENLDNKGRLRVGAAIGVNEIERAKLLVEAGVDVIVLDSAHGHSLNIIRTLKEIKSKMNIDVIVGNVVTEEATKELIENGADGIKVGIGPGSICTTRIVAGVGVPQITAIEKCSSVASKFGIPIIADGGIRYSGDIGKALAVGASSVMIGSILAGTEESPGEKELIGDTVYKYYRGMGSVGAMKSGSGDRYFQEKRPENKMVPEGIEGRVKYKGEMEGVVYQLVGGLRSCMGYLGSASIEELWKKSSYVEITTSGLRESHVHDVEIVKEVMNYSK
CSGID Clone ID	IDP92622
Cloning/expression vector	pMCSG7
Expression host	*E. coli* BL21(DE3) pMAGIC†
Complete amino-acid sequence of the construct produced‡	*HHHHHHENLYFQ^SN*AMGTKNIGKGLTFEDILLVPNYSEVLPREVSLETKLTKNVSLKIPLISSAMDTVTEHLMAVGMARLGGIGIIHKNMDMESQVNEVLKVKNSGGLRVGAAIGVNEIERAKLLVEAGVDVIVLDSAHGHSLNIIRTLKEIKSKMNIDVIVGNVVTEEATKELIENGADGIKVGIGPGSICTTRIVAGVGVPQITAIEKCSSVASKFGIPIIADGGIRYSGDIGKALAVGASSVMIGSILAGTEESPGEKELIGDTVYKYYRGMGSVGAMKSGSGDRYFQEKRPENKMVPEGIEGRVKYKGEMEGVVYQLVGGLRSCMGYLGSASIEELWKKSSYVEITTSGLRESHVHDVEIVKEVMNYSK
Molecular weight (Da)	38459 (40077 with His_6_ tag)
Extinction coefficient§ (*M* ^1^cm^1^)/Abs 0.1% (= 1gl^1^)	0.569 (0.583 with His_6_ tag)

† Kim *et al.* (2011 ▶).

‡ The cloning tag is italicized, with the TEV protease site denoted ‘^’. This construct lacks residues 90134 compared with the sequence deposited in CryptoDB. These residues were replaced with Ser-Gly-Gly (underlined).

§ Calculated using *ProtParam* for the reduced protein sequence (Gasteiger *et al.*, 2005 ▶).

**Table 2:** Crystallization

Method	Sitting-drop vapor diffusion
Plate type	Greiner Bio-One 96-well CrystalQuick standard profile, 3 round wells, flat bottom
Temperature (K)	289
Protein concentration (mgml^1^)	13.4
Buffer composition of protein solution	20m*M* HEPES pH 8.0, 150m*M* KCl, 1.5m*M* TCEP
Composition of reservoir solution	0.1*M* BicineNaOH pH 9.0, 20% PEG 6000
Cryocondition	0.1*M* BicineNaOH pH 9.0, 20% PEG 6000, 15% glycerol
Volume and ratio of drop	1:1 (0.4:0.4l)
Volume of reservoir (l)	135

**Table 3:** Data-collection and processing statistics Values in parentheses are for the highest resolution shell.

No. of crystals	1
Beamline	SBC 19-ID, APS
X-ray wavelength ()	0.97931
Temperature (K)	100
Detector	ADSC Quantum 315r
Crystal-to-detector distance (mm)	300
Total rotation range ()	190
Rotation per image ()	1.0
Exposure time per image (s)	3
Space group	*P*2_1_
Unit-cell parameters (, )	*a* = 89.19, *b* = 91.83, *c* = 92.02, = 90, = 103.75, = 90
Resolution range ()	40.572.05 (2.092.05)
Total No. of observations	349609
No. of unique reflections	89862 (4514)
*I*/(*I*)	12.66 (2.02)
Data completeness (%)	99.9 (99.6)
*R*_merge_ † (%)	14.0 (69.5)
*R*_p.i.m._(*I*) (%)	7.9 (43.4)
Wilson *B* factor (^2^)	27.5
Multiplicity	3.9 (3.3)
Mosaicity ()	0.5031.367

† *R*_merge_ = 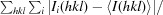 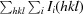, where *I_i_*(*hkl*) is the intensity of the *i*th measurement of an equivalent reflection with indices *hkl*.

**Table 4:** Refinement statistics Values in parentheses are for the highest resolution shell.

Resolution range ()	40.572.053 (2.0762.053)
Reflections used (working/free)	85300/4499 (2661/139)
Data completeness (%)	99.6 (93.0)
*R* factor†/*R*_free_ (%)	18.7/22.9 (26.7/31.8)
Total No. of non-H atoms in asymmetric unit	10414
No. of protein atoms	10312
No. of heteroatoms	
P131	120
IMP	92
Glycerol	24
PEG	14
No. of water molecules	339
R.m.s.d. from ideal geometry	
Bond lengths ()	0.004
Bond angles ()	0.864
Average *B* factors (^2^)	
Overall	36.1
Protein atoms	
All	36.0
Main chain	32.4
Side chain	38.4
Water molecules	40.4
Ligand atoms	
P131	39.4
IMP	26.1
Solvent/cryoprotectant molecules	
Glycerol	62.9
PEG	65.4
Ramachandran statistics (%)	
Most favored	97.3
Outliers	0.0
*MolProbity* scores	
Rotamer outliers	0.9
Clashscore	2.59
Overall score	1.15
PDB code	4rv8

† *R* = 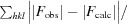 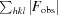, where *F*_obs_ and *F*_calc_ are observed and calculated structure factors, respectively.
